# *Lactobacillus salivarius* and Berberine Alleviated Yak Calves’ Diarrhea via Accommodating Oxidation Resistance, Inflammatory Factors, and Intestinal Microbiota

**DOI:** 10.3390/ani14162419

**Published:** 2024-08-21

**Authors:** Qing He, Sijia Lu, Jia Wang, Chang Xu, Weijie Qu, Shah Nawaz, Farid Shokry Ataya, Yi Wu, Kun Li

**Affiliations:** 1 College of Veterinary Medicine, Nanjing Agricultural University, Nanjing 210095, China; 2 MOE Joint International Research Laboratory of Animal Health and Food Safety, College of Veterinary Medicine, Nanjing Agricultural University, Nanjing 210095, China; 3 College of Veterinary Medicine, Yunnan Agricultural University, No. 452 Feng Yuan Road, Panlong District, Kunming 650201, China; 4 Department of Anatomy, Faculty of Veterinary Science, University of Agriculture, Faisalabad 38000, Pakistan; 5 Department of Biochemistry, College of Science, King Saud University, P.O. Box 2455, Riyadh 11451, Saudi Arabia

**Keywords:** yak calves, diarrhea, *Lactobacillus salivarius*, berberine, microbiota

## Abstract

**Simple Summary:**

Yaks are economically important food animals in the plateau regions of China, but bacterial diarrheal diseases frequently occur, with limited effective treatments. In this study, we investigated the effects of *Lactobacillus salivarius* and berberine on diarrheal yak calves. Our results showed that yaks treated with *Lactobacillus salivarius* and berberine had higher weight growth rates and lower diarrhea scores. Serum analysis indicated that these treatments increased the levels of T-AOC, SOD, GSH-Px, and IL-10 while reducing MDA, TNF-α, IL-1β, and IL-6. Microbiota sequencing identified significant changes in two phyla and twenty-seven genera, including beneficial genera, such as Faecalibaculum and Parvibacter, and harmful genera, like Marvinbryantia and Lachnospiraceae UCG-001. These findings provide novel insights for developing new therapies to combat ruminant diarrhea.

**Abstract:**

Yaks are important food animals in China; however, bacterial diarrheal diseases frequently occur on the plateau, with limited effective therapies. The objective of this research was to evaluate the effectiveness of *Lactobacillus salivarius* (LS) and berberine in alleviating diarrhea in yak calves. For this purpose, eighteen healthy yak calves were divided into control (JC), infected (JM), and treatment (JT) groups. Yaks in the JT group were treated with 2 × 10^10^ CFU/calf *L. salivarius* and 20 mg/kg berberine, and yaks in the JM and JT groups were induced with multi-drug-resistant *Escherichia coli*. The results showed that the weight growth rate in the JM group was significantly lower than that in the JC and JT groups. The diarrhea score in the JM group was significantly higher than that in both the JC and JT groups. Additionally, the contents of T-AOC, SOD, GSH-Px, and IL-10 were significantly lower in the JM group than those in the JC and JT groups, while MDA, TNF-α, IL-1β, and IL-6 were significantly higher in the JM group. Microbiota sequencing identified two phyla and twenty-seven genera as significant among the yak groups. Notably, probiotic genera such as Faecalibaculum and Parvibacter were observed, alongside harmful genera, including Marvinbryantia and Lachnospiraceae UCG-001. Our findings indicate that treatment with *L. salivarius* and berberine significantly reduced diarrhea incidence, improved growth performance, and positively modulated intestinal microbiota, which could provide novel insights for developing new therapies for ruminant diarrhea.

## 1. Introduction

The third pole, the Qinghai-Xizang plateau, is in internal Asia [1] and is characterized by a frigid, anoxic climate with strong ultraviolet radiation [2,3]. In this rugged environment, numerous “boat of the plateau” ruminants—yaks—have been living at altitudes of 3000–5000 m for thousands of years. There are 14 million yaks on the Chinese plateau, which represents 90% of the total yak population globally [4,5]. In the Chinese plateau regions, yaks are economically and religiously important ruminants, providing nourishing meat and milk products, furs, medicinal horns, and dung fuel for native herdsmen [6]. Hence, cattle disease, an especially infectious disease, can negatively affect the yak industry and the local economy on the plateau [4].

Cattle diarrhea caused by intestinal microbes is usually associated with higher morbidity and mortality, especially in calves [7,8]. Infectious diarrhea caused by bacteria, viruses, and parasites is considered a major cause of economic loss in the cattle industry [9]. Besides the mortality caused by serious diarrhea, growth retardation and treatment expenses also reduce the economic benefits of cattle farming, and antibiotic resistance is a growing health concern due to antibiotic overuse [10,11]. Among these microbial pathogens, the opportunistic Gram-negative *Escherichia coli* is a common inhabitant of the gut in warm-blooded hosts and is often detected in many secondary infections. The predominant pathotypes of *E. coli* responsible for diarrhea in yak calves are enterotoxigenic *E. coli* (ETEC), with serotypes O91 and O145 among the most prevalent serotypes [12]. *E. coli* infection in cattle not only causes severe diarrhea but also impedes meat production and trade [13]. Nowadays, antimicrobial resistance is a serious issue for human and animal health [14,15], and many studies have reported antimicrobial resistance in *E. coli* strains on cattle farms [16,17]. Therefore, developing a novel therapeutic method for *E. coli* infection is urgent and important.

Probiotics were first defined as live microbes that promote health when ingested in appropriate amounts by the World Health Organization [18]. Nowadays, probiotics are popularly utilized in food supplements, cosmetics, and for gastrointestinal diseases [19,20]. *Lactobacillus salivarius* is a beneficial bacterium that can mediate gut microbiota and the inflammatory response and maintain the intestinal barrier [21,22]. A previous study confirmed that a strain of *L. salivarius* could alleviate intestinal damage in mice caused by *E. coli* [23]. Berberine is a well-known isoquinoline alkaloid found in plants like *Coptis chinensis, Phellodendron chinense*, and *Berberis aristate*, etc. [24,25]. In traditional Chinese medicine, herbs like Coptis chinensis are used to treat digestive system diseases like diarrhea and jaundice [26]. Previous studies have verified that berberine has anti-bacterial and anti-diarrhea effects [27] and can alleviate colitis and inflammatory bowel disease [28,29]. These studies suggest that *L. salivarius* and berberine could be employed to treat bacterial diarrhea in animals.

The gut microflora is composed of an enormous number of microbes, including bacteria, fungi, protozoa, and viruses [30], which play considerable roles in various physiological processes, such as digestive absorption, nutrition metabolism, and immunity [31]. The balance of microbial immunity is positively related to intestinal homeostasis [32], while microbiome changes are associated with inflammatory bowel disease [33] and irritable bowel syndrome [34]. Previously, microbial dysbiosis has also been reported in diarrheic cattle and yaks [35,36,37]. Until now, little awareness has been mastered regarding the treatment effect of *Lactobacillus salivarius* (LS) and berberine on diarrheic yaks on the plateau. The objective of this research was to evaluate the effectiveness of *Lactobacillus salivarius* and berberine in alleviating *E. coli* (enterotoxigenic *E. coli* (ETEC))-induced diarrhea in yak calves. This was achieved by analyzing their impact on antioxidant capacity, inflammatory factors, and intestinal microbiota. 

## 2. Materials and Methods

### 2.1. Experiment Design

**Figure 1:**
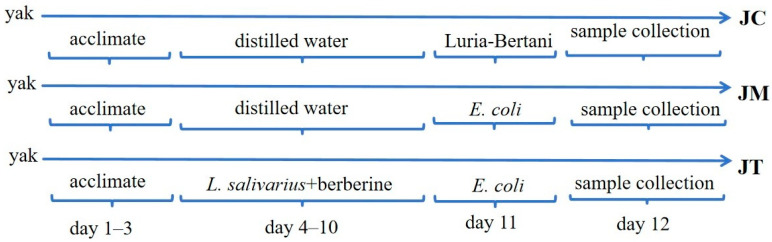
Experimental timeline nd protocol overview.

Eighteen healthy four-month-old male Qinghai–Tibet Plateau-origin yak calves were selected from a yak industrial farm in Linzhou country, China. The calves were housed in individual pens with concrete flooring covered by straw bedding. The housing area was maintained at a temperature of 20 ± 3.5 °C with adequate ventilation, and all calves were given three days to acclimate. Each pen was equipped with a feeding trough and fed on hay and concentrates with pelleted starter feed (265.9 g/kg CP, DM basis) with water supplied *ad libitum*. The pens were refreshed every 3 days, and manure was removed daily to keep the bottoms of the pens dry and clean. For the experimental study, yak calves were randomly grouped into three groups: control (JC), infected (JM), and treatment (JT). From the 4th to the 10th day, yaks in the JT group were treated orally with previously isolated *L. salivarius* (PP859184) at 2 × 10^10^ CFU/calf and 20 mg/kg berberine (Meryer, Shanghai, China. Yaks in the JC and JM groups received an equal volume of distilled water. On the 11th day, ruminants in groups JM and JT were induced with multi-drug-resistant *E. coli* (PP859186) isolated from diarrheal yaks. By contrast, the JC group was induced with an equal volume of Luria–Bertani medium (Solarbio, Beijing-China). On the 12th day, blood and rectal content samples were obtained from all yak calves (Figure 1). Fecal samples were collected directly from the rectum using sterile gloves and stored at −80 °C until analysis. Body weight was measured, and the diarrhea scores of the animals were evaluated. The diarrhea score was assessed on the basis of diarrhea severity as reported in a previous study: normal (0), slightly moist (1), temperately moist (2), loose (3), and watery stools (4) [38].

### 2.2. Detection of Oxidation Resistance and Inflammatory Factors in Yaks

Blood samples from calf veins were centrifuged at 3000× *g* for 15 min to obtain serum for enzyme-linked immunosorbent assay (ELISA). Serum cytokines, including tumor necrosis factor-alpha (TNF-α), interleukin-10 (IL-10), interleukin-6 (IL-6), and interleukin-1 beta (IL-1β), as well as antioxidant indices, malondialdehyde (MDA), superoxide dismutase (SOD), glutathione peroxidase (GSH-px), and total antioxidant capacity (T-AOC), were assessed using commercial assay kits from Jiancheng Bioengineering Institute (Nanjing, China).

### 2.3. Intestinal Microbiota Sequencing of Yak Calves

Eighteen rectal samples from yak calves in the JC, JM, and JT groups were selected for microbial DNA extraction via QIAamp DNA Stool Mini Kit (Qiagen, Hilden, Germany) following the manufacturer’s instructions. The quality and concentration of these extracts were examined using 1% agarose gel electrophoresis and Qubit 3.0 fluorimeter (Thermo Scientific, Waltham, MA, USA), as described in previous studies [39,40]. The V3–V4 zone of the 16S rRNA gene of all yak microbiomes was amplified using the primer pairs 341F/785R [41]. The PCR amplification products were examined to confirm their quality via electrophoresis. Finally, these products were used for library construction using TIANSeq DirectFast Library Kit (Illumina) (Tiangen, Beijing, China) and sent for amplicon sequencing via the Illumina MiSeq platform (Bioyi Biotechnology Co., Ltd., Shanghai, China).

### 2.4. Sequencing Data Analyzing of Yak Calves

The raw data from calf ruminants were processed with cutadapt and QIIME2 (QIIME 2 2020.6.) software to filter sequences and generate an amplicon sequence variant (ASVs) abundance table [42,43]. All yak ASVs were annotated by blasting against the SILVA database (V138). Alpha diversity was analyzed to explore the richness and diversity of microflora within the yak samples by calculating the Shannon, Simpson, ACE, Coverage, Chao1, Observed species, and PD_whole_tree indexes [44]. Beta diversity was assessed to compare the intestinal microbiota composition of different yak groups through principal component analysis [45], principal coordinates analysis [46], and non-metric multidimensional scaling [47]. Significantly different species between calf groups (JC, JM, and JT) were identified using LEfSe and multiple *t*-tests [48,49]. 

### 2.5. Statistical Analysis

Valid difference analysis among yak groups was performed using SPSS version 36.0. The results are presented as means ± standard deviation (SD), and statistical significance was determined at *p* < 0.05. Data normality was assessed using the Shapiro–Wilk test. For normally distributed data, one-way ANOVA was conducted, followed by Tukey’s post hoc test. For non-normally distributed data, log transformation was applied before analysis. Statistical significance was consistently set at *p* < 0.05.

## 3. Results

### 3.1. Lactobacillus salivarius and Berberine Reduced Diarrhea in Yak Calves

**Figure 2:**
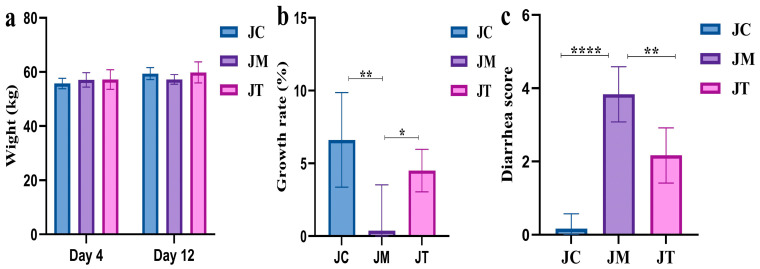
Effects of *Lactobacillus salivarius* and berberine on weight (kg), growth rate (%), and diarrhea score in yak calves. (**a**): Weight gain (kg) in yak calves over the experimental period. (**b**): Growth rates among the different treatment groups. (**c**): Diarrhea severity scores assessed using a standardized scoring system. Significance levels are indicated as follows: **** *p* < 0.0001, ** *p* < 0.01, and * *p* < 0.05. Data are presented as the mean ± SEM (n = 6 per group). These results demonstrate the beneficial effects of *Lactobacillus salivarius* and berberine on reducing diarrhea and promoting weight gain in yak calves.

There were no marked differences in the initial (Day 4) and final (Day 12) body weight between the JC, JT, and JM groups (Figure 2a). However, the weight growth rate in the JM group was notably lower (*p* < 0.01) than that in the JC group, while yaks supplemented with LsB showed a higher growth rate (*p* < 0.05) compared with those in the JM group (Figure 2b). The diarrhea score in the JM group was significantly higher than in the JC (*p* < 0.0001) and JT (*p* < 0.01) groups (Figure 2c).

### 3.2. Lactobacillus salivarius and Berberine Increased Oxidation Resistance and Dropped Inflammation Levels in Ruminants

**Figure 3:**
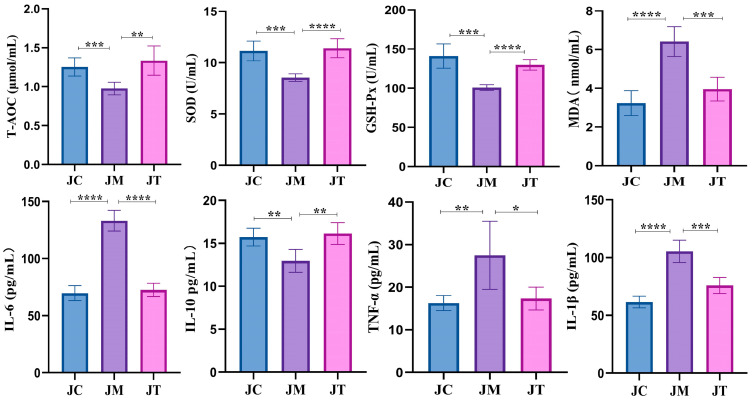
Impact of *Lactobacillus salivarius* and berberine on oxidative stress and inflammatory responses in calves. The graph shows the levels of antioxidant indices, including total antioxidant capacity (T-AOC) (µmol/mL), superoxide dismutase (SOD) (U/mL), glutathione peroxidase (GSH-px) (U/mL), malondialdehyde (MDA) (nmol/mL), along with inflammation levels using serum cytokines, including tumor necrosis factor-alpha (TNF-α), interleukin-10 (IL-10), interleukin-6 (IL-6), and interleukin-1 beta (IL-1β)(pg/mL) in calves treated with *Lactobacillus salivarius* and berberine in the JC, JM and JT groups. Statistical significance is indicated by asterisks: **** *p* < 0.0001, *** *p* < 0.001, ** *p* < 0.01, and * *p* < 0.05. Data are presented as the mean ± SEM with n = 6 for each group. Statistical analyses were performed to determine differences between treatment groups and the control.

Serum analysis showed that the level of T-AOC (*p* < 0.001), SOD (*p* < 0.001), GSH-Px (*p* < 0.001), and IL-10 (*p* < 0.01) were significantly lower in the JM group than in the JC group, while MDA (*p* < 0.0001), TNF-α (*p* < 0.01), IL-1β (*p* < 0.0001), and IL-6 (*p* < 0.0001) were significantly higher in the JM group. Interestingly, calves supplemented with LsB in the JT group had signally higher levels of T-AOC (*p* < 0.01), SOD (*p* < 0.0001), GSH-Px (*p* < 0.0001), and IL-10 (*p* < 0.01) and lower MDA (*p* < 0.01), TNF-α (*p* < 0.05), IL-1β (*p* < 0.001), and IL-6 (*p* < 0.0001) than those in the JM group (Figure 3).

### 3.3. Lactobacillus salivarius and Berberine Mediated the Microbial Community in Yaks in Different Taxa

**Figure 4:**
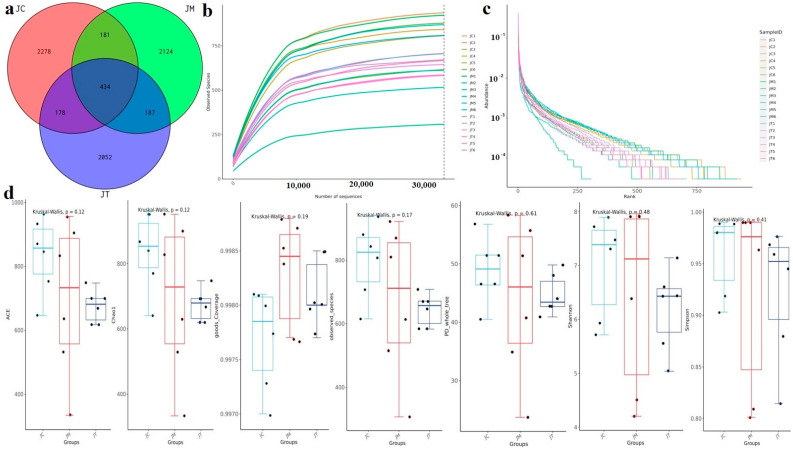
Effects of *Lactobacillus salivarius* and berberine on the microbial community structure and diversity in calves. The panels illustrate different aspects of microbiota analysis. (**a**): Amplicon Sequence Variants (ASVs) Venn diagram showing the overlap and unique ASVs across treatment groups. (**b**): Rarefaction curve depicting the relationship between the number of observed ASVs and sequencing depth, indicating the completeness of sampling. (**c**): Rank abundance plot illustrating the distribution of ASV abundances across different treatment groups. (**d**): Alpha diversity index analysis representing the microbial diversity within each treatment group. Data are shown as the mean ± standard error of the mean (SEM), with n = 6 for each group.

Over 97, 800 (JC), 92, 800 (JM), and 91, 200 (JT) raw sequences were obtained, with more than 94, 200 (JC), 89, 400 (JM), and 88, 300 (JT) filtered sequences achieved in the three calf groups (Supplementary Table S1). These sequences were aligned to 9648 ASVs (JC = 3071, JM = 2926, JT = 3651), and 434 ASVs were found in all calf groups (Figure 4a). The rarefaction curves of all yak samples peaked quickly and remained stable (Figure 4b), indicating high richness in the yak microbiomes. The rank abundance for all ruminant samples was smooth (Figure 4c), indicating high evenness. There was no virtual difference in alpha diversity indices between the groups (Supplementary Table S2, Figure 4d).

**Figure 5:**
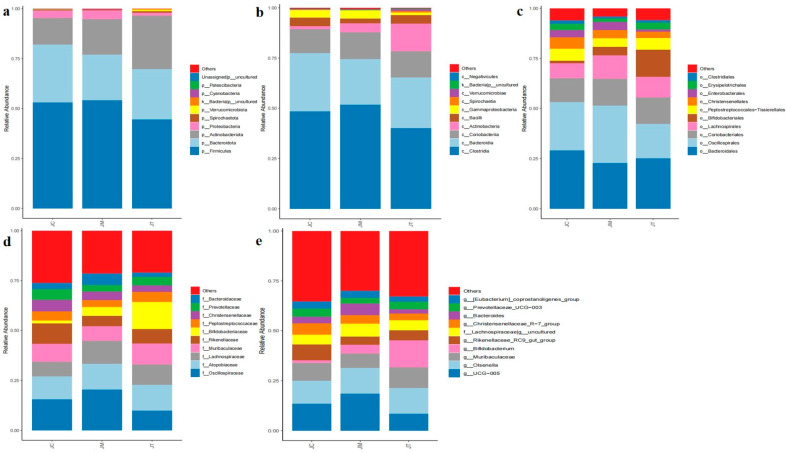
Impact of *Lactobacillus salivarius* and berberine on the microbial community structure in calves at different taxonomic levels. (**a**): Phylum: Distribution of microbial communities at the phylum level across different treatment groups. (**b**): Class: Variation in microbial communities at the class level between treatment groups. (**c**): Order: Representation of microbial communities at the order level in different treatment groups. (**d**): Family: Microbial community structure at the family level across treatment groups. (**e**): Genera: Distribution of microbial communities at the genus level for each treatment group.

**Figure 6:**
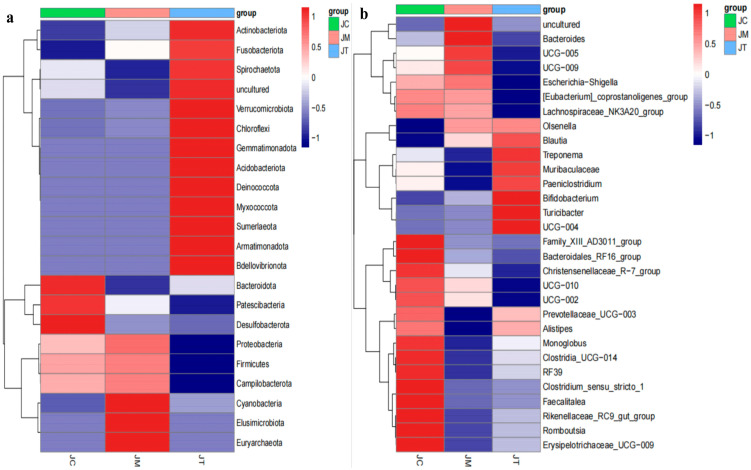
Heat map analysis of the effect of *Lactobacillus salivarius* and berberine on calf microbiota in all groups. (**a**): Phylum. (**b**): Genera. Color intensity represents the relative abundance of each taxon, with warmer colors indicating higher abundance.

At the phylum level, Firmicutes, Bacteroidota, and Actinobacteriota dominated in the JC (53.01%, 29.00%, and 13.29%), JM (54.13%, 22.77%, and 17.86%), and JT (44.54%, 25.18%, and 26.81%) groups (Figure 5a). At the class level, Clostridia, Bacteroidia, and Coriobacteriia were predominant in the JC (48.49%, 29.00%, and 11.93%) and JM (51.68%, 22.77%, and 13.49%) groups, while Clostridia (40.13%), Bacteroidia (25.18%), and Actinobacteria (13.71%) were predominant in the JT group (Figure 5b). At the order level, Bacteroidales, Oscillospirales, and Coriobacteriales were major orders in the JC (28.99%, 24.20%, and 11.93%) and JM (22.75%, 28.68%, and 13.49%) groups, while Bacteroidales (25.14%), Oscillospirales (17.25%), and Bifidobacteriales (13.49%) were major orders in the JT group (Figure 5c). At the family level, Oscillospiraceae (15.54%), Atopobiaceae (11.47%), and Rikenellaceae (10.24%) were the major families in the JC group; Oscillospiraceae (20.32%), Atopobiaceae (12.91%), and Lachnospiraceae (11.55%) were the major families in the JM group; and Bifidobacteriaceae (13.49%), Atopobiaceae (12.90%), and Muribaculaceae (10.40%) were the major families in the JT group (Figure 5d). At the genus level, UCG-005, Olsenellam, and Muribaculaceae were predominant in the JC (13.51%, 11.43%, and 8.95%) and JM (18.52%, 12.79%, and 7.26%) groups, while Bifidobacterium (13.43%), Olsenella (12.83%), and Muribaculaceae (10.40%) were predominant in the JT group (Figure 5e). The heat map showed that the phyla Bacteroidota, Patescibacteria, and Desulfobacterota were abundant in JC, Cyanobacteria, Elusimicrobiota, and Euryarchaeota were abundant in JM, while Actinobacteriota, Fusobacteriota, Spirochaetota, Verrucomicrobiota, Chloroflexi, Gemmatimonadota, Acidobacteriota, Deinococcota, Myxococcota, Sumerlaeota, Armatimonadota, and Bdellovibrionota were abundant in the JT group (Figure 6a). At the genera level, Family_XIII_AD3011_group, Bacteroidales_RF16_group, Christensenellaceae_R−7_group, Monoglobus, Clostridia_UCG−014, RF39, Clostridium_sensu_stricto_1, Faecalitalea, Rikenellaceae_RC9_gut_group, Romboutsia, and Erysipelotrichaceae_UCG−009 were abundant in the JC group; Bacteroides was abundant in the JM group; and Bifidobacterium, Turicibacter, and UCG−004 were abundant in the JT group (Figure 6b).

### 3.4. Distinguished Species among Yak Calves in Different Groups

**Figure 7:**
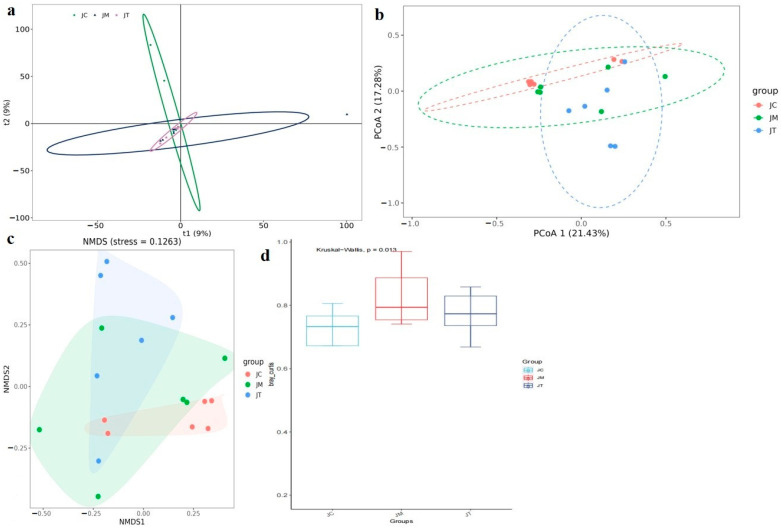
Beta diversity analysis of calves among different groups. (**a**): Principal component analysis (PCA). (**b**): Beta diversity principal coordinate analysis (PCoA). (**c**): NMDS. (**d**): Kruskal−Wallis. Beta diversity analysis revealed significant differences between the JC, JM, and JT groups (Figure 7a–c), confirmed by the Kruskal−Wallis analysis indicating significant differences (*p* < 0.05) between the three calf groups (Figure 7d).

**Figure 8:**
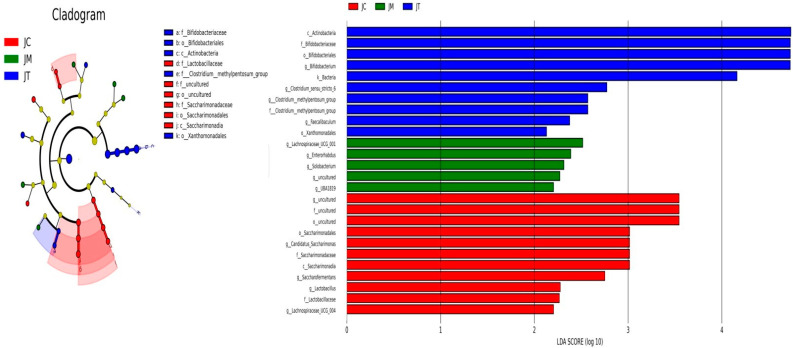
Linear discriminant analysis effect Size (LEfSe) on selected marker species among the JC, JM, and JT groups. Only lineages with least discriminant analysis (LDA) values of >2.0 are displayed. Group differences are represented by the color of the most abundant lineages (blue: surface; green: DCM; and red: deep). Cladogram indicating the phylogenetic distribution of the lineages associated with the JC, JM, and JT groups. Circles indicate phylogenetic levels (from domain to class) in reverse order. The diameter of each circle is proportional to the abundance of the given taxon.

Beta diversity analysis revealed significant differences between the JC, JM, and JT groups (Figure 7a–c), confirmed by the Kruskal−Wallis analysis indicating significant differences (*p* < 0.05) between the three calf groups (Figure 7d). LEfSe analysis identified that Actinobacteria (*p* < 0.05), Bifidobacteriales (*p* < 0.05), Xanthomonadales (*p* < 0.05), Bifidobacteriaceae (*p* < 0.05), Bifidobacterium (*p* < 0.05), Clostridium sensu stricto 6 (*p* < 0.05), the Clostridium methylpentosum group (*p* < 0.05), and Faecalibaculum (*p* < 0.01) were significantly higher in the JT group (Figure 8); Saccharimonadia (*p* < 0.05), Saccharimonadales (*p* < 0.05), Saccharimonadaceae (*p* < 0.05), Lactobacillaceae (*p* < 0.05), Candidatus Saccharimonas (*p* < 0.05), Saccharofermentans (*p* < 0.05), Lachnospiraceae UCG 004 (*p* < 0.05), and Lactobacillus (*p* < 0.05) were significantly higher in the JC group; and Enterorhabdus (*p* < 0.05), Lachnospiraceae UCG 001 (*p* < 0.05), UBA1819 (*p* < 0.05), and Solobacterium (*p* < 0.01) were significantly higher in the JM group. 

**Figure 9:**
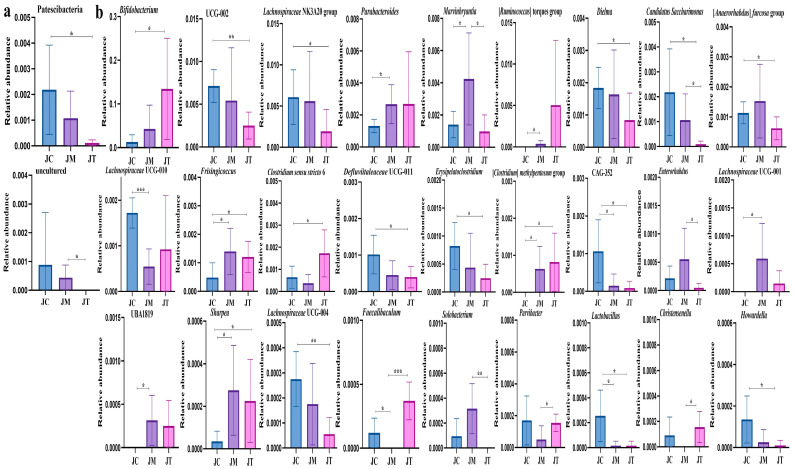
Identification of marker species among different yak groups using multiple *t*-tests. Identification of significant marker species among different yak groups at various taxonomic levels using multiple *t*-tests: (**a**) Phylum: Marker species identified at the phylum level across different yak groups. (**b**) Genera: Marker species identified at the genus level among the yak groups. The statistical analysis highlights species with significant differences among the JC, JM, and JT groups, providing insight into the distinct microbial profiles of each group. Statistical significance is indicated by asterisks: * *p* < 0.05, ** *p* < 0.01, *** *p* < 0.001.

Multiple *t*-tests revealed that the phylum Patescibacteria (*p* < 0.05) was significantly lower in the JT group than in the JC group, and an uncultured phylum was markedly lower in the JT group than the JM group (*p* < 0.05) (Figure 9a). Compared with calves in the JC group, Bifidobacterium (*p* < 0.05), Frisingicoccus (*p* < 0.05), Clostridium sensu stricto 6 (*p* < 0.05), (Clostridium) methylpentosum group (*p* < 0.05), and Sharpea (*p* < 0.05) were significantly higher in the JT group, while UCG-002 (*p* < 0.01), Lachnospiraceae NK3A20 group (*p* < 0.05), Dielma (*p* < 0.05), (Anaerorhabdus) furcosa group (*p* < 0.05), Defluviitaleaceae UCG-011 (*p* < 0.05), Erysipelatoclostridium (*p* < 0.05), CAG-352 (*p* < 0.05), Candidatus Saccharimonas (*p* < 0.05), Lachnospiraceae UCG-004 (*p* < 0.01), Lactobacillus (*p* < 0.05), and Howardella (*p* < 0.05) were significantly lower in the JT group. The genera Parabacteroides (*p* < 0.05), Marvinbryantia (*p* < 0.05), (Ruminococcus) torques group (*p* < 0.05), Frisingicoccus (*p* < 0.05), (Clostridium) methylpentosum group (*p* < 0.05), Lachnospiraceae UCG-001 (*p* < 0.05), UBA1819 (*p* < 0.05), and Sharpea (*p* < 0.05) were significantly higher in the JM group than in the JC group, while Lachnospiraceae UCG-010 (*p* < 0.001), CAG-352 (*p* < 0.05), Faecalibaculum (*p* < 0.05), and Lactobacillus (*p* < 0.05) were markedly lower in the JM group. The abundance of Marvinbryantia (*p* < 0.05), Enterorhabdus (*p* < 0.05), Candidatus Saccharimonas (*p* < 0.05), and Solobacterium (*p* < 0.01) were markedly higher in the JM group than in the JT group, while Faecalibaculum (*p* < 0.001), Parvibacter (*p* < 0.05), and Christensenella (*p* < 0.05) were significantly lower in the JM group (Figure 8b).

## 4. Discussion

Yaks are pivotal livestock in the Chinese plateau region, crucial for producing high-quality meat and dairy products that have gained popularity in recent decades. However, cattle, especially calves, are susceptible to diarrhea caused by *E. coli*, which seriously threatens their health and productivity [50,51].

As observed in previous studies, *E. coli* induces significant infection in yaks [52], resulting in the higher diarrhea scores and impaired weight gain observed particularly in the JM group. Interestingly, supplementation with LsB in yak calves led to higher growth rates and mitigated weight impairment. To elucidate the underlying mechanisms of LsB on diarrheal animals, we investigated serum antioxidant capacity and inflammation in calves. Markers such as T-AOC, SOD, GSH-Px, and MDA are well-established indicators of antioxidant enzyme activity, reflecting the oxidative stress status of the host [53]. In our study, *E. coli* infection induced oxidative damage characterized by decreased T-AOC, SOD, and GSH-Px and increased MDA levels in the JM group, consistent with findings in bacteria-infected animals [54,55]. Conversely, treatment with LsB reversed these trends in the JT group, indicating that LsB enhances the antioxidant capacity of ruminants. TNF-α, IL-1β, and IL-6 are critically inflammatory factors [56], while IL-10 plays a crucial role in limiting proinflammatory responses and inflammatory injury [57,58]. In this study, *E. coli* infection resulted in elevated levels of TNF-α, IL-1β, and IL-6, accompanied by decreased IL-10 levels in the JM group, consistent with findings in animals induced with bacterial and Lipopolysaccharide challenges [59,60]. Interestingly, LsB administration reduced the levels of these inflammatory factors and increased IL-10 levels, suggesting that LsB alleviates intestinal damage by modulating inflammatory responses in yaks.

Subsequently, we conducted micro-community sequencing and obtained 1,879,179 raw and 1,809,878 filtered sequences, resulting in alignment to 9648 ASVs, with 434 ASVs shared across yak calves. There was no remarkable difference observed in the alpha diversity index, which partially aligns with findings in *E. coli*-challenged animals [61,62] but contrasts with a study by Wang et al., 2024 [63]. Analysis at different taxonomic levels revealed that *E. coli* infection altered the microbiome of yaks, while LsB partially restored the microbial structure. For instance, at the phylum level, Firmicutes (JC = 53.01%, JM = 54.13%, JT = 44.54%), Bacteroidota (JC = 29.00%, JM = 22.77%, JT = 25.18%), and Actinobacteriota (JC = 13.29%, JM = 17.86%, JT = 26.81%) were dominant across all yak groups, albeit with varying relative abundances. The Firmicutes/Bacteroidota ratio was notably higher in the JM group (2.48) than in the JC (1.83) and JT (1.77) groups; this is a commonly used indicator of dysbiosis [64]. Our findings suggest that LsB mitigates intestinal damage by modulating the gut microbiota.

We further detected marked differences in bacterial composition among yak groups, identifying two phyla and twenty-seven genera that showed significant variations. Among these, Marvinbryantia, known for its higher abundance in depressive mice [65] and animals with intestinal damage induced by high-fat feeds [66], as well as Lachnospiraceae UCG-001, which was observed at higher levels in ulcerative colitis mice [67], were found to be less abundant in JT yaks, suggesting that LsB could potentially reduce these pathogenic bacteria. Similarly, UBA1819 and Solobacterium, genera associated with various diseases [68,69], and Sharpea, which was found at higher levels of abundance in Cryptosporidium-infected pigs [70], were also less abundant in JT yaks, indicating a potential role of LsB limiting the colonization of these pathogenic bacteria. Previous research has linked a lower abundance of Lachnospiraceae UCG-010 with chronic kidney disease in pigs [71], while Christensenella is recognized as an important genus in healthy hosts [72]. Faecalibaculum, a probiotic genus negatively associated with inflammation [68], and Parvibacter, which produces short-chain fatty acids [34], were found at higher abundances in JT calves, suggesting that LsB may alleviate intestinal injury by promoting the proliferation of these genera in plateau ruminants. Our results indicate that LsB restores the balance of the intestinal microbiota in yaks. 

## 5. Conclusions

In conclusion, this study investigated the role of *Lactobacillus salivarius* and berberine in the prevention and treatment of yak calves’ diarrhea. The results were consistent with our expectation that *Lactobacillus salivarius* and berberine can mitigate bacteria diarrhea in yaks by regulating oxidation resistance, inflammatory factors, and microbiota. Moreover, these interventions increase the abundance of probiotic genera (Faecalibaculum and Parvibacter) and decrease harmful genera (Marvinbryantia and Lachnospiraceae UCG-001). Our results provide novel insights for developing new therapies for ruminant diarrhea.

## Data Availability

Sequence data from yak calves were stored in the NCBI database under accession number PRJNA1131151.

## Supplementary Materials

The following supporting information can be downloaded at: https://www.mdpi.com/article/10.3390/ani14162419/s1. Table S1. The sequencing data of yak calves in groups JC, JM, and JT. Table S2. Statistical analysis of alpha diversity index in yak calves in group JC, JM and JT.
