# Supplementary material for: Lactobacillus salivarius and Berberine Alleviated Yak Calves’ Diarrhea via Accommodating Oxidation Resistance, Inflammatory Factors, and Intestinal Microbiota
Source: Animals (Basel). 2024 Aug 21;14(16):2419. doi: 10.3390/ani14162419 (PMC11350718; doi:10.3390/ani14162419)
Supplement: Supplementary file 1 [file animals-14-02419-s001.zip › animals-3128443-Supplementary Materials.pdf]

animals

Table S1. The sequencing data of yak calves in groups JC, JM, and JT.

Samples	input	filtered	percentage of input passed filter	denoised	merged	percentage of input merged	non-chimeric	percentage of input non-chimeric
JC1	99352	96062	96.69	91784	69458	69.91	41549	41.82
JC2	109459	105493	96.38	100279	69246	63.26	37039	33.84
JC3	97822	94227	96.32	89914	62777	64.17	33302	34.04
JC4	120221	115307	95.91	111008	80300	66.79	47203	39.26
JC5	116831	112069	95.92	107964	84299	72.15	55097	47.16
JC6	111940	107673	96.19	103225	75891	67.8	46581	41.61
JM1	104264	100277	96.18	95794	67677	64.91	35121	33.68
JM2	106010	101676	95.91	99642	83668	78.92	50779	47.9
JM3	93169	89793	96.38	86920	68950	74.01	37547	40.3
JM4	109079	104942	96.21	101452	79256	72.66	40017	36.69
JM5	101451	97652	96.26	93144	62961	62.06	36615	36.09
JM6	92879	89408	96.26	85016	55607	59.87	33847	36.44
JT1	100667	97301	96.66	93528	72140	71.66	44794	44.5
JT2	101230	97624	96.44	93687	67841	67.02	38447	37.98
JT3	102913	99278	96.47	95365	71830	69.8	41041	39.88
JT4	113329	109434	96.56	106201	84710	74.75	48120	42.46
JT5	107275	103288	96.28	99484	73996	68.98	39333	36.67
JT6	91288	88374	96.81	84410	62474	68.44	36374	39.85

Table S2. Statistical analysis of alpha diversity index in yak calves in group JC, JM and JT.

Sample	observed species	ACE3	Chao1	Shannon	Simpson	PD_whole_tree	goods_Coverage
JC1	707	752.6312476	770.1613	5.9298	0.9187	46.93173608	0.9973
JC2	937	965.1359321	959.78	7.8937	0.9898	56.80288398	0.998
JC3	843	868.9825394	867.5854	7.475	0.9814	51.30620356	0.9981
JC4	807	845.0485874	839.5111	7.3017	0.9789	46.18643074	0.9977
JC5	615	645.567384	640.2133	5.7129	0.903	40.4818273	0.9981
JC6	881	932.5401036	943.1923	7.7148	0.9875	51.56555965	0.997
JM1	922	956.0757733	952	7.8669	0.9887	55.73965277	0.9977
JM2	307	333.857157	333.0909	4.2027	0.8011	23.69208673	0.9987
JM3	515	531.4679972	528.9811	4.5064	0.8086	34.92396381	0.9988
JM4	613	633.8221523	629.1096	6.3803	0.9635	40.71163766	0.9985
JM5	870	905.8905394	900.163	7.9626	0.991	51.35164816	0.9977
JM6	810	831.8543191	827.6667	7.8653	0.9901	58.33963585	0.9984
JT1	708	747.7930338	747.5132	5.552	0.8795	47.99404207	0.9977
JT2	672	694.3033313	693.25	7.1439	0.9764	42.79686813	0.9985
JT3	646	667.9729926	666.9016	6.6044	0.9451	42.62606514	0.9985
JT4	583	616.8214842	619.2459	5.0392	0.8144	40.89370768	0.998
JT5	585	619.8788653	620.0952	6.4696	0.9679	44.05175374	0.998
JT6	668	700.9170349	691.3152	6.3919	0.9592	49.81188097	0.998
